# Extract from Dr. E. D. Fenner’s Report on Salicine in the Treatment of Intermittent Fever, at the New Orleans Hospital

**Published:** 1846-02

**Authors:** 


*Extract from Dr. E. D. Fenner's report on Salicine in the treatment of Intermittent fever, at the New Orleans Hospital.*—Total amount of medicine given in the 20 cases; salicine, ℥vii, ʒvi, ℈ii; piperine, ʒi ℈ii; quinine ʒiv.

Largest amount given in any case; salicine, ʒvii; piperine, ʒiss; quinine. ʒiii, ℈i.

Smallest amount given in any case; salicine, ʒi, grs. x; piperine, grs. xii; quinine, ℈ii.

Average amount of salicine to each patient ʒiii, grs. viii.

It was first tried alone in all the 20 cases.

It succeeded when given alone, in 11 cases.

It failed when given alone, in 9 cases.

Of these, it succeeded when combined with piperine, in 4 cases.

Quinine had to be resorted to in 6 cases.

Average time of sickness previous to treatment, 7½ days.

Do " " under treatment 6½ days.

Greatest number of paroxysms, after salicine was commenced, 6 days,

Smallest " " " " " 0 days,

Average " " " " " 9 "

There were one or more paroxysms after salicine was commenced, in 14 cases.

There were none, in 6 cases.

I am informed by the Apothecary of the Hospital that the cost of salicine was $2 00 per oz. Consequently, the value of the amount used in these 20 cases, (say 7¾ oz.,) was about $15 50; and of the average amount given to each patient, (say ʒiii,) 75 cents. The article appeared to be fresh and genuine.

I prescribed it in the forms of solution, powder and pill; in doses varying from *five grains, to one drachm;* and at intervals of from one hour to twelve.

The general effects of the remedy appeared to be *tonic and diaphoretic.* The appetite and strength were generally improved, and the sweating was profuse. I observed *no unpleasant effect* that I could attribute to the remedy. Where it failed *to do good, it did no harm.*

*Comparative statement of* 20 *cases of Intermittent Fever treated with the sulphate of quinine.*

After the foregoing observations on the use of salicine were completed, I resolved to note 20 cases of intermittent fever treated mainly with quinine; with the view of ascertaining the relative *efficacy* and *cost* of the two remedies. Twenty recent admissions were taken throughout the wards of the Hospital, and of course under the care of different physicians. Upon inquiry I found that no two of them administer the remedy alike; some of them prescribe it in large doses, and at long intervals; others, the reverse; some give it alone; others, in combination with blue mass, opium, or morphia. As minute notes were taken of these 20 cases, as of the preceding; but for fear of wearying the reader, I will only give the *results,* and the conclusion to which they brought me.

Whole amount of quinine used in the 20 cases, about ℥iss.

Largest amount given in any case, ʒi.

Smallest amount given in any one case, grs. xviii.

Average amount of quinine given to each patient, grs. xxxvi.

It was given combined with sulph. morphia, grs. xii. to gr. ½ in 2 cases.

It was given combined with ext. opii, grs. xvii, to gr. i, in 4 cases.

" " with blue mass; grs. vi, to grs. x, in 1 case.

" " alone in 13 cases.

*All the cases were promptly cured.*

Average time of sickness before admission; 10 days.

" " after 4 "

Greatest number of paroxysms in any case after taking quinine, 2 days.

Smallest " " " " " 0 days.

Average number of paroxysms, 35 "

There were one or more paroxysms after taking quinine, in 10 cases.

There was none, in 10 cases.

The cost of quinine was $3 25 pr. oz, consequently the value of the whole amount used in these 20 cases, [say ½ oz.] was $4 87½, the average amount, (say 36 grains,) about 26 cents.

Candour compels me to state that the cases treated by salicine were generally more severe, than those treated by quinine; it will be recollected that one of them was so malignant as to be with difficulty saved by upwards of ʒiii of quinine, after having previously taken ʒiiss of salicine. The salicine cases occurred chiefly in the months of June and July, when intermittents usually assume their worst form ; the quinine cases all occurred about the first of October, when the disease is generally mildest. These circumstances are worthy of grave consideration, lest we be enduced to underrate the actual virtues of salicine. However, taking the two sets of cases as they are presented to us in the foregoing comparative statement, and reviewing the effects of the two remedies in their various combinations beforementioned, we are brought to the conclusion that *the average amount of quinine required to cure* 20 *cases of intermittent fever, and costing* 25 *cents, is fully three times as efficacious as the average amount of salicine required in a like number of cases, and costing* 75 *cents.*

The comparison made in this instance cannot, however, be considered a perfectly fair one ; but when the foregoing reports are taken in conjunction with others that will doubtless be made from the medical department of the Army, they may aid in leading us to a proper estimate of the virtues of salicine. How many of the foregoing cases would have had their paroxysms broken up merely by *the change of residence, and attention to regimen, without any medicine whatever*, must remain a matter of conjecture. My own opinion is, there would have been a goodly number. Hence the importance of exercising a sound judgment and careful observation in regard to the action and comparative value of medicines.—*New Orleans Medical and Surgical Journal, Nov.* 1845.

